# Towards Energy Efficient Home Automation: A Deep Learning Approach

**DOI:** 10.3390/s20247187

**Published:** 2020-12-15

**Authors:** Murad Khan, Junho Seo, Dongkyun Kim

**Affiliations:** School of Computer Science and Engineering, Kyungpook National University, Daegu 41566, Korea; mkhan@knu.ac.kr (M.K.); junhoseo@knu.ac.kr (J.S.)

**Keywords:** smart homes, energy management, Internet of Things, machine learning

## Abstract

Home Automation Systems (HAS) attracted much attention during the last decade due to the developments in new wireless technologies, such as Bluetooth 4.0, 5G, WiFi 6, etc. In order to enable automation as a service in smart homes, a number of challenges must be addressed, such as fulfilling the electrical energy demands, scheduling the operational time of appliances, applying machine learning models in real-time, optimal human appliances interaction, etc. In order to address the aforementioned challenges and control the wastage of energy due to the lifestyle of the home users, we propose a system for automatically controlling the energy consumption by employing machine and deep learning techniques to smart home networks. The proposed system works in three phases, (1) feature extraction and classification based on 1-dimensional Deep Convolutional Neural Network (1D-DCNN) which extract important energy patterns from the historic energy data, (2) a load forecasting system based on Long-short Term Memory (LSTM) is proposed to forecast the load based on the extracted features in phase 1 and (3) a scheduling algorithm based on the forecasted data obtained from phase 2 is designed to schedule the operational time of smart home appliances. The proposed scheme efficiently automates the smart home appliances to consume less energy while adapting to the lifestyle of smart home users. The validation of the proposed scheme is tested with a number of simulation scenarios incorporating datasets from authentic data sources. The simulation results show that the proposed smart home automation system can be a game-changer in fulfilling the energy demands of the home users without installing renewable and other energy sources in the future.

## 1. Introduction

**Figure 1:**
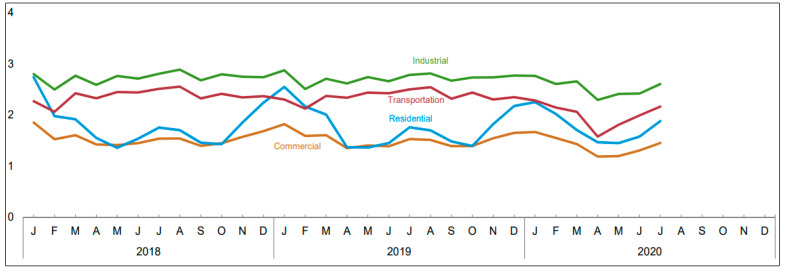
Energy consumption in various sectors from January 2018 to July 2020 (Unit: QuadrillionBtu) [1].

The future of autonomous smart homes mainly depends on the efficient processing and data analysis of energy and load data. Recently, we have witnessed an increase in energy demand for smart homes and cities. According to the current literature, every year an excessive amount of energy is consumed in the residential sector as shown in Figure 1 [1]. A huge peak can be seen in the winter season as the home users consume much energy for heating systems. Among this energy consumption, a huge amount of energy is wasted in the residential sector due to the inefficient human interaction with the electronic appliances of a smart home. Similarly, the lack of processing energy and other relevant data leads to poor performance in handling the energy crisis. The IoT can play a major role in generating data for detecting patterns that can be used to design efficient systems for tackling the energy crisis in the future smart homes, smart buildings, etc. Similarly, the current smart home meters play an important role in collecting energy data from smart home appliances and could be interrogated for future use. Similarly, the introduction of cloud and edge computing makes it easier for researchers to process data in real-time with powerful machine learning algorithms, quantum, and super-computing. In addition, the introduction of 5G networking technology makes it easier for transferring huge amounts of data with high speed and bandwidth. Similarly, the recognition of patterns of a particular purpose also needs powerful feature extraction and segmentation techniques. The research in the fields of implementing sensor networks within smart homes, smart cities, etc., can produce data that leads to Human Activity Recognition (HAR) techniques. The HAR lays down a platform for researchers to build predictive methods that can help in eradicating the excessive usage of energy and other relevant energy issues that exist in a smart home environment. Besides, the data from various activities such as the interaction of a smart home user with an appliance in different times of day, etc., can be stored and processed in offline time for various purposes such as scheduling the home appliances, notifying the home user if the energy of a smart home exceeds a limit, etc. Such processing of data can be further used for the well-being of humans in various sectors such as building construction with efficient energy usage, smart parking with electric charging facilities, identifying gas leakage, etc.

To design an efficient and real-time energy management system, the data obtained from smart home appliances are further classified into different groups. This classification helps in reducing computation time in preprocessing steps and applying machine learning models to the data. However, a number of challenges present in the classification of data such as identifying the sensors attached to high-priority appliances, real-time analysis of data, grouping similar types of home appliances together, and so on. Similarly, traditional methods are mostly occupied by supervised learning such as Support Vector Machine (SVM), Random Forest (RF), etc. [2,3]. Therefore, processing the data generated from heterogenous HAR sources with supervised learning techniques requires a huge amount of prior labeling of data. Later on, Deep Neural Networks (DNN) and Convolutional Neural Networks (CNN) are widely adopted for the classification and processing of HAR data for extracting important features. However, such techniques require labeled and annotated datasets for efficient processing. Therefore, the researchers introduce techniques based on attention mechanisms to boost the performance of the DNN and CNN for weakly annotated and labeled data [4,5]. The attention-based mechanisms are mostly used for image classification and semantic segmentation. Therefore, using such mechanisms for real-time unsupervised HAR data may result in poor classification and feature extraction. Similarly, the classification mainly depends on the features selected for training. However, selecting the best features for training is a challenging and computationally expensive job. A number of mechanisms such as transform coding [6], Fourier transformation based symbolic representation [7], etc., were recently introduced for HAR data feature extraction. However, such schemes exhibit heuristic nature and do not provide task dependency. In addition, these schemes require high computational cost without improving the classification performance. Besides, such schemes require multi-dimensional data for training purposes. On the other hand, those devices which are used for collecting energy data such as smart meter always generate a 1-dimensional time sequence of data which also requires further programming to classify data based on appliances usage, etc. Finally, the current research has many limitations before applying the DNN and CNN methods for feature extraction from energy data. However, if somehow the data is classified with the DNN method, another challenge arises in designing autonomous smart homes is to predict the energy consumption of smart homes at a particular time of the day. In this regard, a number of machine and deep learning algorithms based on Artificial Neural Network (ANN) is proposed in the literature. However, the ANN always produce significant results for short term prediction. In the case of long-term prediction such as predicting the energy consumption of a smart home for an entire day, month, and even a year, the ANN performs inefficiently. Therefore, to design an autonomous smart home with a long-term prediction of the energy consumption of appliances, a machine learning method such as an LSTM algorithm is needed. The current literature consists of a number of approaches predicting the short-term energy consumption of home appliances [8]. However, such schemes perform inefficiently in the case of long-term predictions. The long-term prediction of energy data is widely ignored in the current literature. Therefore, the applications of log-term predictions cannot be used for scheduling the appliances for a longer time. In this regard, the long-term prediction models are presented for analyzing historic energy data using the LSTM model [9,10]. However, these models have still limitations: they are used for specific scenarios, the testing datasets were limited to a specific set of residents, etc. Therefore, it is necessary to use the full power of the Bi-directional LSTM (BLSTM) model for forecasting with high accuracy.

In this research work, we proposed an architecture of an autonomous smart home system based on deep learning models for feature extraction and classification, prediction, and scheduling of home appliances. A 1D-DCNN is first used to analyze the smart home data and extract important features of energy consumption at various times of the day. Based on the extracted energy values, a BLSTM model is used to predict the energy consumption for the next month. Though the data is available in the time-series sequence, and it is difficult to model it via ANN, the BLSTM shows significant improvement in long-term predictions. The predicted energy consumption is further optimized for as minimum as possible energy consumption using the proposed scheduling technique based on Reinforcement Learning (RL). The RL performs the scheduling of agents attached to each appliance of a smart home. The agents of RL communicate with each other whenever a user performs an action of switching ON and OFF an appliance. For instance, if switching on an appliance increases the energy consumption of a smart home from a defined threshold, then the agent of the respective appliance communicates with the rest of the agents in the smart home to perform an action of either switching off or lowering the power level of an electronic appliance. Finally, an extensive set of simulations is performed to check the accuracy and performance of the proposed scheme with authentic datasets. The proposed achieve high accuracy in the case of predicting the activities. Similarly, the scheduling of operational time of appliances is presented to the smart home user to perform and operate the appliances in a schedule that consumes as much as less energy.

## 2. Related Works

Every year an excessive amount of energy gets wasted due to the improper use of electronic appliances in smart homes and buildings. In this regard, the literature consists of a number of literature to handle the improper use of electricity in smart homes and cities with the scheduling of operational time of appliances, etc. However, such schemes either focused on specific scenarios or target one particular aspect of smart homes or cities. In addition, a generic system dealing with all parameters involved in the energy consumption of smart homes is rarely addressed in the literature. The interaction of smart home users with electronic appliances requires attention by implementing machine deep learning models over the historic data. However, the machine and deep learning models always require extensive computation and show inefficient performance in real-time computing. Furthermore, human nature is extremely dynamic, and modeling human behavior towards appliances’ interaction requires a large number of parameters to be tuned with great precision and care. The Wireless Sensor Network (WSN) can play a vital role in scheduling the appliances with programmed human behavior. For instance, a sensor can be programmed to switch off and on an electronic appliance upon the interaction of a home user. In this regard, the literature consisted of a number of schemes to handle the interaction of home users in smart homes using WSN technology [11,12,13].

Building an autonomous smart home requires a huge amount of changes to the current infrastructure of smart homes [14,15,16]. The proposed scheme in [14] presented an infrastructure of controlling the things inside a smart home by dividing the things into two groups, i.e., active and passive. These things are control with the help of agents attached to each appliance. The proposed system is specifically designed for elderly people. Thus, using such a system for young and adults may require extra care because their frequency of using things is relatively high for elderly people. The system is tested for accuracy of detecting things; however, some other important parameters, such as classification of activities in a day and nighttime and human appliances interaction are not considered for a completely autonomous system. Similarly, a system presented in [16], where the energy requirements of the smart homes are fulfilled with renewable energy sources. The smart homes communicating with the smart grid for energy demand and the smart grid continuously provides/supplies the energy from energy storage systems or renewable energy systems. The energy supply from a renewable energy system always suffers from uncertainties if the weather condition is not favorable. Therefore, a machine learning technique is required to control the uncertainties of renewable energy sources in such a scenario, which is missing in the proposed work. Autonomous smart homes require a system consisting of multiple phases from classifying human activities to the scheduling of home appliances. Although in the literature there exist a number of mechanisms based on machine learning to improve the working of classifying multidimensional activities’ data. However, everything inside a smart home cannot be automated because it would require a huge amount of changes to the existing smart home infrastructure. For instance, to make an air-conditioning system automatic is easy compared to a chair or any other mechanical thing. Therefore, researchers need to work on those things which could give maximum benefits to society, such as automation. In this regard, a Home Energy Management System (HEMS) is proposed to handle the excessive cost of electricity consumption using optimization methods based on genetic algorithm in [17]. The proposed scheme is tested with distributed power systems and an electric vehicle. In both the objective function presented in the said work, the authors skipped the user comfort level dependency on the proposed system. In such an environment, where a home user sets a demand for electricity from a smart grid, and if somehow the smart grid is unable to fulfill the demand, the user discomfort level rises. Therefore, incentive-based schemes are widely presented in the current literature to reduce the discomfort level of the user in smart home networks [18,19]. In [18], the authors presented the home user with incentives based on shifting and scheduling the appliances from peak hours to non-peak hours. The electricity firms share the compensation in the form of financial rewards in the monthly billing with those home users who successfully schedule many of the task-based appliances in the non-peak hours. However, user behavior is always dynamic, and it may not be possible for every home user to schedule the appliances in the non-peak hours. Similarly, if there are multi-users available in a smart home, the performance efficiency greatly reduced. Similarly, in the case of [19], the authors suggested offering incentives to those home users who participated in the scheduling of appliances pattern offered by the electricity generation firm. However, the user needs to share the load pattern and other relevant details with the firm to better decide the scheduling pattern. Furthermore, it is recommended by the firms to the consumers to use photovoltaic power generation and batteries. In addition, such systems increasing the cost of obtaining the required hardware and maintenance.

The data generated by an electronic appliance is normally available in the form of a single dimension (1D), i.e., time-series data. The classification of time series data is extensively studied in the existing literature [20,21]. However, these schemes mainly classify the data for short-term forecasting purposes. For instance, the authors proposed a system for Demand Response (DR) based on reinforcement learning and artificial neural network in [21]. The authors proposed an hour-ahead DR algorithm based on forecasting of energy prices. The proposed system efficiently solves the problem of energy demand in advance; however, the input to the system is taken from the energy supply companies which is a challenging job in real-time. A similar scheme is presented in [22], where the authors proposed to forecast the electric load of a feeder, substations, or transformers based on DL methods. The proposed DL based forecasting incorporates a number of environmental and load parameters for feature selection. After the selection of important features, the DL method is tested in a number of scenarios with various energy datasets. The proposed method successfully classified the features. However, the results are not tested for energy consumption after and before applying the scheduling of operational time of the smart home appliances.

Short-term electric load prediction is widely studied in the current literature [23,24]. These schemes mainly employing machine learning techniques, such as ANN, DNN, Support Vector Machine (SVM), K-means, Wavelet Neural Network (WNN), etc. Similarly, these models predict and forecast the electric load of a smart home, smart grid, smart building, etc. with high accuracy and precision [23]. The scheme presented in [23], forecast the short-term load based on a hierarchical structure. The child nodes, such as end-user customers are further divided into regular and irregular customers. Both the regular and irregular customers’ energy patterns are forecasted separately to reduce the burden on the entire network. However, these schemes require a huge amount of data for training and also expensive frequent computation. As human behavior is dynamic and every human being’s interaction with electronic appliances is different from each other. Therefore, relying on short-term forecasting is always better for one particular home user data, however, cannot be transferred to another home user. Similarly, if there exists more than one home user, then even predicting short-term load in the form of regular and irregular child nodes could result in poor performance. In this regard, the long-term load prediction and forecasting models are employed to overcome the challenges present in the short-term forecasting models [25,26]. In many of these research works; the LSTM model is widely adopted for forecasting long-term load. The LSTM model operates using three different gates, i.e., input, output, and forget gate as discussed in Section 3.4 for processing the data. However, the LSTM model always performs better if trained with a huge amount of data as discussed in [25]. Furthermore, there is a need for extensive tuning of hyper-parameters. Therefore, the LSTM model is computationally costly and may still result inefficient if trained on less amount of data. In the case of the scheme proposed in [26], the authors forecast the energy data with LSTM for hour-ahead scenarios. LSTM is used for forecasting which has certain limitations of controlling error once the data is outputted. Therefore, it is necessary to use other variations of LSTM or ANN and DNN for forecasting of time series data. Similarly, if the LSTM model is employed to data that is already pre-processed or refined with another machine learning algorithm then it might work well. Thus, in the current literature, the LSTM model is used in concatenation with other machine learning models to produce better results [27,28]. In this regard, a hybrid LSTM and CNN model is presented to forecast the photovoltaic power consumption in [28]. The proposed approach extracts two types of features from the data, i.e., LSTM and CNN are used to extract the temporal and spatial feature information, respectively. As the hybrid model extracts rich information resulting in high accuracy in terms of detection and significantly less amount of error loss. However, to design an autonomous smart home it is necessary to classify the features and later on used it for forecasting loads. The current literature consisting of such hybrid models that could help in achieving partial autonomy; however, the challenge of creating an autonomous home remains undiscovered. The forecasted load can help in scheduling the home appliances in advance. In order to achieve less energy consumption, the literature consists of a number of schemes for scheduling operational time of home appliances [29,30]. For instance, in [30] a grey wolf and crow search optimization technique is used to schedule the home appliances. The proposed scheme incorporates a real-time price signal for electricity cost reduction, minimizing user discomfort level, and reduces the peak to average ratio. The scheduling of appliances significantly reduces the energy consumption; however, if the scheduling algorithm is applied to forecasted data, the results would be better. The forecasted energy consumption can better help in scheduling the operational time of appliances in advance. The scheduling of operational time of home appliances in real-time is a challenging job. Therefore, the researchers tried to overcome this issue by implementing online scheduling mechanisms, such as mixed-integer linear programming, game-theoretic models, binary backtracking search algorithm, etc. [31,32]. However, deciding the scheduling of appliances always depends on the historical data which is rarely discussed in these schemes. Furthermore, such schemes can perform better in a specific environment and therefore cannot be employed in generic environments. Finally, the schemes used in the related work section are summaries in the following Table 1.

## 3. Proposed System

### 3.1. Problem Statement

The future autonomous smart homes require a number of machine learning methods running in parallel to perform different tasks such as feature extraction from the historic data, classification of data, and forecasting the load for scheduling and other relevant purposes. However, merging and concatenating all these steps logically is a difficult and challenging job due to the following reasons.

- The energy demand is increasing rapidly and in the future, the energy demands can be fulfilled by installing new energy-generating technologies such as nuclear plants and greenhouses. However, installing nuclear plants generate radiations, and the greenhouses result in extreme carbon dioxide emission which results in affecting the community.
- The projected energy consumption demands may increase in the future. A huge difference between energy demand and supply may result in increasing the cost of energy.
- Apart from generating energy by installing new energy technologies there exists other methods and guidelines of using and interacting with appliances. However, such techniques exhibit many issues, such as improper scheduling, modeling human behavior, and interaction towards appliances, difficulty in modeling human behavior, etc.
- Limitations of the machine and deep learning techniques in processing energy data for prediction and forecasting. Furthermore, the current research work mainly focuses on short-term forecasting of energy consumption and cost; however, long-term forecasting is mainly ignored due to the limitations of using ANN and similar learning models.
- The CNN and other relevant models are effective in modeling high dimension, i.e., two or more dimensional and uses huge data for feature extraction; however, this is less effective in the case of feature extraction in 1-Dimensional (1-D) times series data.
- The current home appliances are not intelligent and there is a lack of communication among appliances.

Keeping in mind the above challenges, we designed an autonomous smart home based on the historic data of the energy consumption of the appliances. The proposed scheme extracts and classifies important features from the data using the CNN model. The data values are then fed into a BLSTM model which forecasts the energy consumption values for the next month. Finally, the forecasted energy consumption is used to schedule the operational time of the home appliances using the QL model to consume as less as possible energy.

### 3.2. Contribution of the Proposed Scheme

In this section, we highlighted the main contributions of the system as follows:

- A classification of the energy data into different groups based on the time of a day is carried out. This would reduce the processing of the data in later stages.
- The energy data is time-series data with a single dimension, we, therefore incorporate and proposed an approach based on BLSTM. The later approaches mainly used ANN and other deep learning techniques which require a huge amount of data for training.
- The scheduling of home appliances is carried out using the Q-learning reinforcement algorithm on the forecasted data. This enables a home user to decide the future scheduling of home appliances based on the results of the proposed scheduling.
- The energy of the home appliances is significantly reduced and an autonomous home system is achieved incorporating various deep learning and artificial intelligence models.

### 3.3. Overview of the Proposed System

**Figure 2:**
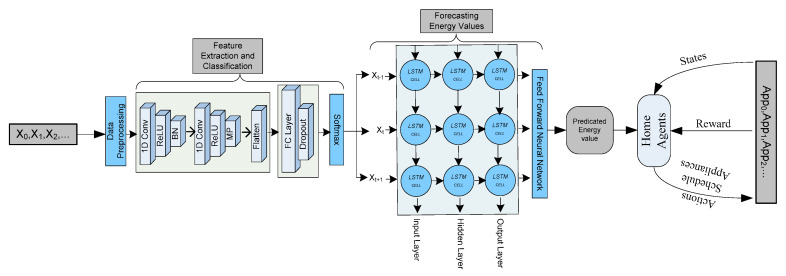
The architecture of the proposed scheme.

The architecture of the proposed system is shown in Figure 2. The proposed system consisted of three main steps, i.e., (1) feature extraction and classification of energy data using 1D-DCNN, (2) forecasting of energy data using BLSTM, and (3) scheduling of operational time of electronic appliances using QL model as shown in Figure 2. As a human interacts with various electronic appliances during the entire course of the day, resulting in generating a sequence of energy consumption values. The energy consumption values do not contain enough information apart from the energy load of the entire home. Thus, it is important to extract important features and classify them according to the load consumption at various times of the day. The electric load values are then passed to a BLSTM model to forecast the load for the next day (24 h) and month. The reason for forecasting based on the classified data is the actual data does not contain important information and, thus, it will affect the forecasting process. In the forecasting of electric load for the next day process, the BLSTM inputs a number of parameters from the feature classification phase as well as temperature information of the same location from where the dataset is collected. The reason for processing the temperature information is that it directly affects the energy consumption of the homes as shown in Figure 1. After forecasting the electric load for the next day, we proposed a scheduling process based on the QL model to schedule the electronic appliances with minimum energy consumption and high user comfort. Finally, we visualize the results of various phases to better show the strength of the proposed scheme.

### 3.4. Preprocessing Phase

As we know that the smart meter collects energy consumption data from the sensors attached to various home appliances. Thus, there are possibilities that the data contain ambiguities and erroneous data due to climate change, faulty meter problems, etc. In order to remove the noise and erroneous data from the datasets, a smoothing filter technique presented in [33] is adopted in this research work. The proposed smoothing filtering technique is widely used for filtering and refining the time series data. After performing the smoothing process, we labeled the data in order to fit it to the CNN based feature extraction process. The classes of the labels are designed based on the energy data generated by a smart meter at a particular time of the day. As our final goal is to predict the future energy consumption values and schedule the operational time of the electronic appliances based on them, therefore, it is important to extract the most relevant features in the feature extraction phase. However, extracting features from time-series data is a challenging job; therefore, it is necessary to label the data with great care. The labels used in our proposed feature extraction and classification are shown in Table 2. Initially, we have tested the model with a smaller number of labels and later increases to check for the best number of labels for 1D-DCNN.

**Figure 3:**
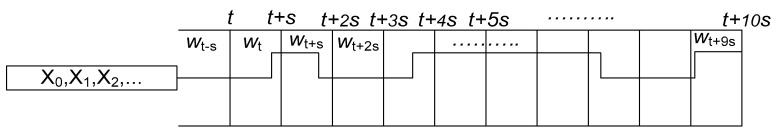
A fixed sliding window technique.

After performing the labeling process, the data segmented using a Fixed Sliding Time Window (FSTW) technique. The proposed FSTW technique shifts the sliding window with a fixed length of time to generate various segments. These segments are then isolated for further processing in the feature extraction phase. The FSTW technique operates using two parameters, i.e., a shift and window size parameter represented with s and w, respectively, as shown in Figure 3. As shown in Figure 3, the data from a smart meter is divided into various segments employing the FSTW technique to efficiently feed into the feature extraction algorithm.

### 3.5. Feature Extraction and Classification Phase

Feature extraction is a challenging job in the case of times series data. However, due to the proposed segmentation and preprocessing of data with important labels, it is now easy to extract important features. The features from each time sliding window are extracted using the 1D-DCNN. In order to define the 1D-DCNN, we start with the preparation of input data which is a 1D matrix with the window size w and m as a dimension of the sensor reading. The actual representation of the sensor reading is represented with the dimension vector as shown in the following equation [34].

(1) $$S_{1}^{T}=\left\{x_{1},x_{2},x_{3},\cdots ,\;x_{T-1},\;x_{T}\right\}$$

where *x* is the load value from a sensor at time *t*.

Further, each segment is passed to a 1D-DCNN as shown in Figure 2. A 1D-DCNN is used to extract various features from the preprocessed energy data. An instance of input x data R further consisting of a timestamp value and a feature set P. The input instance *x* is passed to a 1D-DCNN with a filter represented with f. A feature map is constructed at each layer of CNN using the following equation.

(2) $$x_{k}^{i}=b_{k}^{i}+\overset{N_{i-1}}{\underset{m=1}{\sum }}conv1D\left(f_{mk}^{i},o_{m}^{i-1}\right)$$

where $x_{k}^{i}$ is an input to a neuron of CNN, $b_{k}^{i}$ represents the bias of the *k*^th^ neuron at layer *i*, and $o_{m}^{i-1}$ is the output of the i−1 neuron. The weight matrix $f_{mk}$ represents the kernel from the previous neuron, i.e., i−1 to the current neuron *i*. The conv1D represents the convolution function which operates over input data using a filter of variable size.

After performing the convolution operation at each neuron, the result is passed to the Rectified Linear Unit (ReLU) activation function and Maximum Pooling (MP). The ReLU represented with σ outputs the input value directly if it is positive or changes it to positive in the case of the proposed scheme if it is negative. The ReLU is used because many of the values of energy data may be recorded zero whenever none of the appliances is operating at home. The output of the ReLU is computed bypassing the output represented with $CO_{i}$ from the convolution layer to the ReLU as shown in the following equation.

(3) $$O_{{\mathrm{ReLU}}_{i}}=\sigma \left(CO_{i}\right)$$

After the ReLU operation, the feature map is passed to the Batch Normalization (BN) to standardize the input to a layer. Inside a convolution function, the BN is used in the first part and the MP layer is added in the second part to downsample the feature maps. A flatten layer is applied to create a single long feature vector to pass it to a Fully Connected (FC) layer. A DropOut (*DO*) mechanism is applied to avoid overfitting in the final output. Finally, the classification of the data is performed with the help of a SoftMax activation function represented with $SM_{i}$ on the output of the dropout layer as shown in the following equation.

(4) $$SM_{i}=\;\frac{e^{O_{i}^{DO}}}{\sum_{z=1}^{N}e^{O_{z}^{DO}}}$$

### 3.6. Load Forecasting Using LSTM

The ANN is widely used for forecasting short-term loads; however, employing ANN for a long-term load is considered inappropriate. Due to this weakness of using ANN for long-term load predictions, we employed the BLSTM to predict and forecast the long-term load based on the output obtained during the feature extraction phase. However, forecasting does not depend only on the energy consumption values obtained at different times of the day. Therefore, we integrate a number of parameters to optimizes the forecasting of the data. It is worth mentioning that selecting inputs for the LSTM algorithm is a tedious and challenging job. Therefore, in this research, we give great attention to selecting the best inputs based on the correlations of input to the output [35,36]. Apart from the guidelines mentioned in the aforementioned sources, we also tuned the values of the input parameters during the simulation process. The input parameters used in the proposed approach are given in Table 3. These parameters are obtained from the datasets discussed in Section 4.1. The BLSTM model is trained with 80% of the data from the datasets and 20% is used for testing purposes. However, the data are composed of instances of electricity consumption for approximately two years; therefore, there is a possibility that the results generated for a particular time may incorporate wrong instances. Thus, we split the data based on the date of the data generated into two main seasons, i.e., (1) hot and (2) cold. Then the average results of both seasons were presented in Section 4.2.2.

The information of the inputs 1–8 is extracted from the datasets during the feature extraction and classification phase. However, the information of temperature is not available in the datasets, therefore the information of the temperature is feed into the proposed BLSTM system from a temperature dataset available with the same datasets [37]. Furthermore, during the research, we have noticed that using the information of the temperature in the prediction process significantly improves the forecasting process.

**Figure 4:**
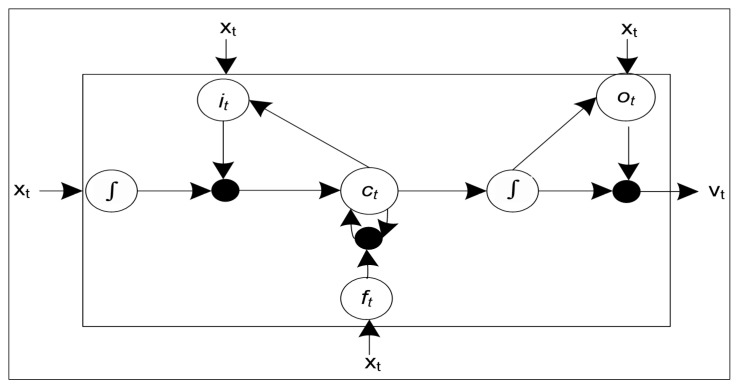
An LSTM cell structure.

This input is now passed to the BLSTM model as shown in Figure 2. The BLSTM model entirely depends on the BLSTM cell which lieu in the heart of the BLSTM model. This cell takes the input value *x_t_* and a hidden vector *v_t_*_−1_ from the previous time step and then produces an estimated output *o_t_* along with a memory vector *m_t_*. The structure of the LSTM cell is shown in Figure 4. Furthermore, the entire computation is represented using the following equations.

(5) $$f_{t}=\sigma (W_{f}\cdot \left[h_{t-1},x_{t}\right])+b_{f}$$

(6) $$i_{t}=\sigma (W_{i}\cdot \left[h_{t-1},x_{t}\right])+b_{i}$$

(7) $$c_{t}=\sigma (W_{c}\cdot \left[h_{t-1},x_{t}\right]+)b_{c}$$

(8) $$o_{t}=\sigma (W_{o}\cdot \left[h_{t-1},x_{t}\right])+b_{o}$$

(9) $$f_{t}=o_{t}\cdot tanh\left(c_{t}\right)$$

As the above equations presented that the state of a BLSTM cell is cleared, written, and accessed with gates called forget (*f_t_*), input (*i_t_*), and output (*o_t_*), respectively. The W and b represent the weights and the biases that are learned during the training phase. The BLSTM architecture has consisted of a number of layers and each layer outputs a unique predicted value. Therefore, it is important to concatenate all the outputs in a single value. In the proposed work, the concatenation is carried out using the feedforward neural network. The feedforward neural network maps the final output from many BLSTM layers to a single value as shown in Figure 2. The final predicted value is generated for a duration of 24 h with a time difference of 30 min. Furthermore, the accuracy of the predicted values is tested with the error predicting methods discussed in the results and discussion section.

### 3.7. Operation Time Scheduling of Home Appliances

**Figure 5:**
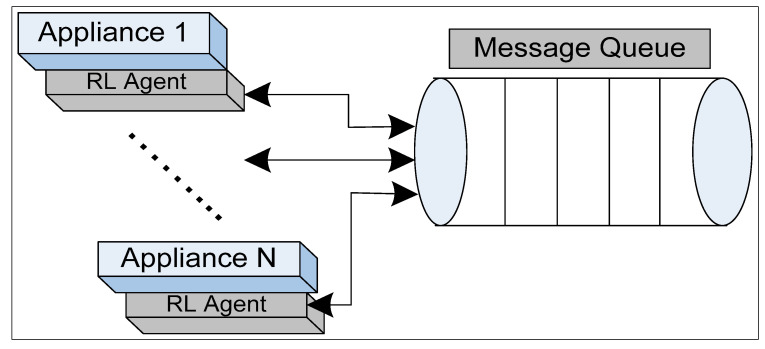
Message passing among appliances’ agents.

The predicted values are passed to the proposed scheduling system designed for scheduling the operational times of smart home appliances. The proposed scheduler is based on the RL algorithm. The RL agents operate on the phenomenon of performing an action a on an environment and receive a reward r. If the reward is high, the RL agents perform a similar action again and again finally reaching the goal state g. In the proposed scheduling scheme, we attached an RL agent to each appliance to perform a suitable action based on the predicted value. Similarly, if the action is not suitable, the RL agent communicates with the rest of the RL agents to adjust their slots accordingly. For instance, if performing an action leads to high energy consumption, the agents of the respective appliance communicate with the rest of the agents to change their power levels to low consumption mode. The communication among agents is enabled with the help of a message-passing service. Each agent in further attached to a queue of messages. In the queue, each agent pushes a message, and the rest of the agents are allowed to read the message to perform the necessary actions. Our aim is to enable a home appliance to perform an action intelligently and seamlessly. The communication of agents via message queue service is shown in Figure 5.

## 4. Performance Evaluation

In this section, we provide a detailed discussion of the experimental analysis of the proposed scheme along with a discussion on the datasets used for the experimentation.

### 4.1. Datasets Description

The proposed scheme is tested on the publicly available energy consumption of home appliances datasets obtain from various smart meters installed in the London city, United Kingdom [37]. The datasets consisted of a number of smart meter data; however, we used the dataset for training and testing obtained from the smart meters of house number MAC00050. The datasets of MAC00050 consists of a record of energy values of 23,782 instances for a period of 12/8/2011 to 4/17/2013. The dataset presented energy values for a 30 min sampling rate over a period of around 2 years. However, in the case of testing the proposed feature extraction and classification, we have selected the first 5000 instances. Out of those 5000 instances, 80% of the data is used for testing, and 20% for training. Further, the energy consumption is given in a unit of kWh which is a standard unit of measuring energy consumption. The weather information is used from the same datasets available in [37].

### 4.2. Performance Evaluation

In this section, we performed an extensive set of experiments to validate the performance of the proposed scheme. Similarly, this section is further breakdown into the following subsections to elaborate the proposed idea.

#### 4.2.1. Analysis of 1D-CNN for Feature Extraction and Classification

The performance of the first part, i.e., feature extraction and classification based on 1D-CNN is tested with *precision*, *recall*, *F-measure*, and *accuracy* coefficient. These metrics are mathematically presented in the following equations.

(10) $$precision=\frac{TP}{\left(TP+FP\right)}$$

(11) $$recall=\;\frac{TP}{\left(TP+FN\right)}$$

(12) $$F-measure=\;2\times \frac{precision\;\times recall}{\left(precision+recall\right)}$$

(13) $$accuracy=\frac{TP+TN}{\left(TP+FP+FN+TN\right)}$$

where *TP*, *FP*, *TN*, and *FN* represent the true positive, false positive, true negative, and false negative, respectively.

The experimentation of the feature extraction and classification process is carried out on the data obtained from the smart meter number MAC00050. The experiments are compared with a simple 1D-CNN as shown in Table 4. The 1D-DCNN performs better when the number of samples is more in a window. However, deep convolution outperforms the 1D-CNN by incorporating more number of layers and fewer samples of a dataset. Furthermore, the energy datasets that are currently available have energy consumption records per hour or a day. Therefore, we proposed 1D-DCNN to achieve high accuracy compare to 1D-CNN.

#### 4.2.2. Analysis of Forecasting Using BLSTM

The performance of the second part, i.e., load forecasting using the BLSTM model is evaluated with the Mean Square Error (MSE), Root Mean Square Error (RMSE), Mean Absolute Error (MAE), and Mean Bias Error (MBE) as shown in Table 5. The last metrics, i.e., MAPE and MBE produce better comparison results of actual and predicted values. Furthermore, the first three methods are widely adopted in the case of regression models.

**Figure 6:**
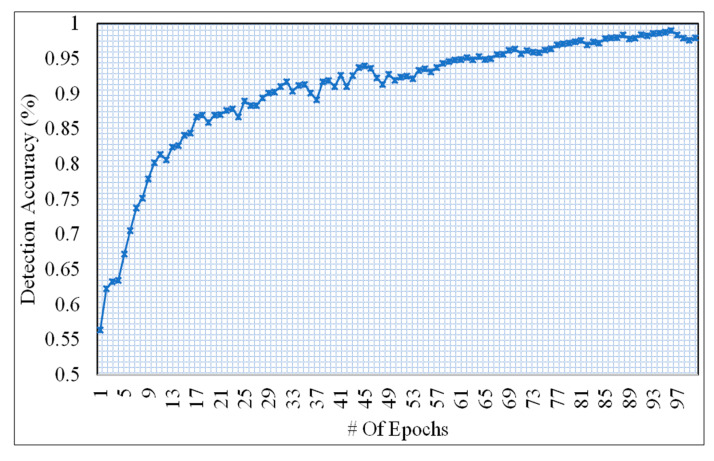
Prediction accuracy of BLSTM for 100 epochs.

During the simulation, we have noticed that the performance of the BLSTM model is affected by the hypermeters exist in the data. Therefore, those parameters are optimized and tuned using the hold out procedure. The experiments are performed with 100 epochs on data of 5000 instances from the dataset of the smart meter number MAC00050 as shown in Figure 6. However, during the experiments, we have noticed that after 70 epochs the results changes with quite marginal values. As we can see along the y-axis the prediction accuracy significantly increases.

**Figure 7:**
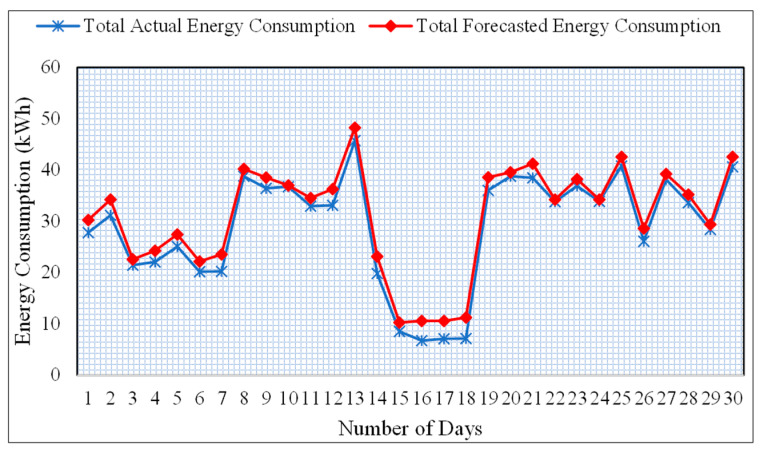
Comparison of forecasted and actual energy consumption of smart meter # MAC00050.

The predicted and actual energy consumption of a one-month smart meter data from meter # MAC00050 is shown in Figure 7. The energy consumption of predicted output is compared with the actual values for the 1-month duration with a sampling rate of 1-value of energy consumption for the entire day. The reason for comparing the 1-month values is to provide a clear visual understanding of the results. As we can see that the forecasted energy consumption is quite similar to the actual energy consumption. In the case of the proposed scheme, the MAE and MAPE are significantly less compared to the LSTM model. Based on these results, we can easily conclude that the BLSTM algorithm performs better in the case of forecasting time series data. It is worth mentioning here that the performance of BLSTM can be further improved by adding more layers. However, in that case, the cost of computation may increases.

**Figure 8:**
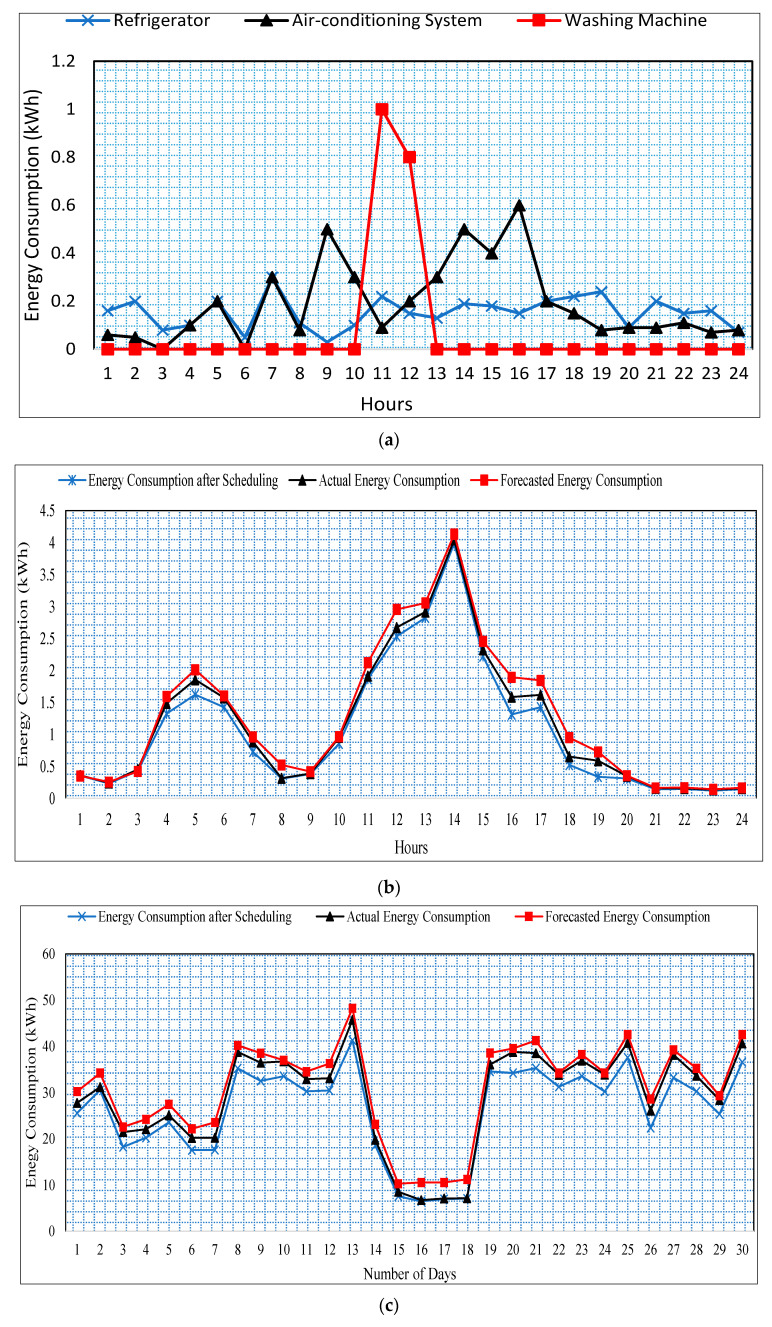
(**a**) Energy consumption of refrigerator, air-conditioning system, and washing machine, (**b**) Energy Consumption comparison of actual, forecasted, and after scheduling for one day (**c**) Energy Consumption comparison of actual, forecasted, and after scheduling for a period one month.

Finally, the actual and forecasted energy consumption is compared with energy consumption after applying the proposed scheduling algorithm based on QL. The scheduling is applied to the forecasted data to test the result of the scheduling process on the forecasted data. This will help the home user to select the best scheduling of appliances in advance. As we can see, the proposed scheme efficiently schedules the appliances by attaching agents with each appliance of the smart home. Furthermore, the proposed message-passing system adds intelligence to the appliances by communicating with each other. Up to our knowledge, this is the first-ever work in which the appliances are provided with the intelligence. This work can help the organization and firms in designing intelligent appliances in the future. Similarly, the lifestyle of the home users can be controlled with the proposed HAS. As the current literature shows that an excessive amount of energy-wasting every year due to the lifestyle of the home users such as most of the time they are leaving home without turning off unnecessary appliances, forget to turn off appliances after use, etc. As illustrated in Figure 1, the residential sector accounts for a major portion of the total energy consumption and, therefore, is important to take care of the wastage of energy in the residential sector. In this regard, the proposed scheduling algorithm performs significantly better compared to actual energy usage as shown in Figure 8a–c. In the case of Figure 8a, the energy consumption of various appliances is shown after the proposed scheduling is applied to the smart home network. The energy consumption of the appliances is significantly reduced as we can see most of the electronic appliances are moved from peak hours into non-peak hours. The washing was originally scheduled in the peak hour time; however, applying the proposed scheme the washing machine is scheduled to non-peak hours. In the case of Figure 8b, the energy consumption per day is shown compared to actual, forecasted, and energy consumption after the appliance operational schedule. As we can see in Figure 8b, the proposed scheme saves approximately 2.223 kWh of energy per day. Similarly, in the case of Figure 8c, the proposed scheme saves around 78.79 kWh of energy per month. This saving of energy significantly reduces the energy consumption of smart homes. Resulting in reducing the energy cost for the entire month and a year.

## 5. Conclusions

In this article, we proposed an autonomous smart home system based on machine and reinforcement learning. The proposed scheme works in three phases, i.e., (1) feature extraction and classification based on 1D-DCNN, (2) electric load forecasting based on BLSTM with a number of parameters from authentic datasets, and (3) scheduling of operational time of appliances based on QL. The proposed scheme efficiently controlled the wastage of energy in smart homes with less effect on the smart home user’s comfort level. Furthermore, the proposed scheme adopts the lifestyle of the home user incorporating the power of reinforcement learning into appliances. The intelligent appliances perform automatic actions based on the user input, i.e., switching on and off an appliance. Similarly, the proposed scheme is tested in a number of scenarios for all three phases and the results obtained show that energy consumption can be significantly reduced in a smart home. The proposed system could be used by the electricity firms and companies for full filing the energy demand with new ways of generating electricity. In the future, we are planning to test the system on real-time testbeds to validate the experimental analysis with real-time experiments.

## Figures and Tables

**Table 1:** Summary of the main schemes discussed in the literature.

Reference	Algorithm	Energy Consumption	User Comfort	Advantages	Disadvantages
**[14]**	Multi-Agent	No	Yes	Efficient automatic control of things for elderly people Multi-agent architecture	Inefficient Real-time performance Data analytics needed
**[16]**	Demand-Response	Yes	No	Optimal coordination among energy systems Reduces energy consumption cost	Renewable energy sources are used without considering the climate variation
**[17]**	Genetic Methods	Yes	Partially satisfied	A two-stage optimization achieved The cost of electricity is significantly reduced	The environmental parameters are not considered User dissatisfaction increases electricity demands does not satisfied
**[18]**	Demand-Response	Yes	No	Efficiently shifting task-based appliances to non-peak hours Electricity cost is significantly reduced	A comparison with the ToU method is not enough The dissatisfaction increases with reducing electricity consumption
**[21]**	ANN + RL	Yes	Partially satisfied	The appliances are categorized into various groups Forecasting of electricity price Efficient decision making using the RL AI model	Reducing the electricity cost increases the complexity of the system by switching appliances into non-peak hours User discomfort may increase if the demand changes with environmental conditions
**[23]**	WNN	Yes	No	The load is forecasted at different levels Efficient short-term load forecasting	May suffer from long-term dependencies User satisfaction at different levels is not addressed
**[26]**	LSTM + RNN	Yes	No	Long-term load forecasting Computation time reduces for offline training	The limitation of using LSTM is ignored User dissatisfaction may arise if the energy demand is not fulfilled by the grid
**[28]**	LSTM + CNN	No	Partially satisfied	Efficient photovoltaic power prediction A hybrid model is used to enhance the prediction accuracy	The model does not explain the uncertainties caused due to environmental conditions User satisfaction is not explained if the prediction goes wrong
**[30]**	GWCSO	Yes	Yes	The cost of electricity is significantly reduced Scheduling of appliances	The scheduling is only tested with the air-conditioning system load The scheduling is also tested with non-shiftable appliances which may increase user dissatisfaction
**[Proposed Scheme]**	1D-DCNN + BLSTM + RL	Yes	Yes	The energy consumption is significantly reduced The user discomfort is reduced The cost of electricity is reduced with the reduction in energy consumption	The Q-learning is used which incur its heuristic limitation of slow convergence due to a high number of samples

**Table 2:** Class labels generated for 1D-DCNN.

Time	Daytime	Label Class
09:00:00 PM–5:00:00 AM	Night	1
05:00:00 AM–11:00:00 AM	Morning	2
12:00:00 PM–3:00:00 PM	Afternoon	3
3:00:00 PM–08:00:00 PM	Evening	4

**Table 3:** Input parameters to the BLSTM model.

Input Index	Parameters Description
1	Energy Consumption on a time of the day (i.e., morning, evening, etc)
2	Time of the day (with 30-min gap)
3	Day of the week (1–7)
4	Holiday
5	Energy demand on Weekends (Saturday and Sunday)
6	Energy demand in last day
7	Energy demand in last week
8	Energy demand in last month
9	Average temperature of the day
10	Average temperature of the month

**Table 4:** Analysis of 1D-DCNN compare to 1D-CNN.

Model	Precision (%)	Recall (%)	F-Measures (%)	Accuracy (%)
1D-CNN	75	75	74.82	75.03
1D-DCNN	91	91	90.67	90.41

**Table 5:** Analysis of BLSTM with LSTM for forecasting time series energy data.

Model	MSE	RMSE	MAE	MBE
LSTM	0.3012	0.5452	0.3122	0.03650
BLSTM	0.2822	0.5102	0.2920	0.03220
